# Alternative Splicing of the Angiogenesis Associated Extra-Domain B of Fibronectin Regulates the Accessibility of the B-C Loop of the Type III Repeat 8

**DOI:** 10.1371/journal.pone.0009145

**Published:** 2010-02-10

**Authors:** Elisa Ventura, Francesca Sassi, Arianna Parodi, Enrica Balza, Laura Borsi, Patrizia Castellani, Barbara Carnemolla, Luciano Zardi

**Affiliations:** 1 Laboratory of Recombinant Therapeutic Proteins, CBA – Advanced Biotechnology Centre, Genoa, Italy; 2 Laboratory of Cell Biology, Istituto Nazionale per la Ricerca sul Cancro, Genoa, Italy; 3 Laboratory of Immunology, Istituto Nazionale per la Ricerca sul Cancro, Genoa, Italy; University of Florida, United States of America

## Abstract

**Background:**

Fibronectin (FN) is a multi-domain molecule involved in many cellular processes, including tissue repair, embryogenesis, blood clotting, and cell migration/adhesion. The biological activities of FN are mediated by exposed loops located mainly at the interdomain interfaces that interact with various molecules such as, but not only, integrins. Different FN isoforms arise from the alternative splicing of the pre-mRNA. In malignancies, the splicing pattern of FN pre-mRNA is altered; in particular, the FN isoform containing the extra-domain B (ED-B), a complete FN type III repeat constituted by 91 residues, is undetectable in normal adult tissues, but exhibits a much greater expression in fetal and tumor tissues, and is accumulated around neovasculature during angiogenic processes, thus making ED-B one of the best markers and targets of angiogenesis. The functions of ED-B are still unclear; however, it has been postulated that the insertion of an extra-domain such as ED-B modifies the domain-domain interface and may unmask loops that are otherwise cryptic, thus giving FN new potential activities.

**Methodology:**

We used the mAb C6, which reacts with ED-B containing FN, but not with ED-B-free FN and various recombinant FN fragments containing mutations, to precisely localize the epitopes recognized by the mAb C6.

**Conclusion:**

We formally demonstrated that the inclusion of the alternatively spliced angiogenesis-associated ED-B leads to the unmasking of the FNIII 8 B-C loop that is cryptic in FN molecules lacking ED-B. Thus, the mAb C6, in addition to providing a new reagent for angiogenesis targeting, represents a new tool for the study of the potential biological functions of the B-C loop of the repeat FNIII 8 that is unmasked during angiogenic processes.

## Introduction

**Figure 1:**
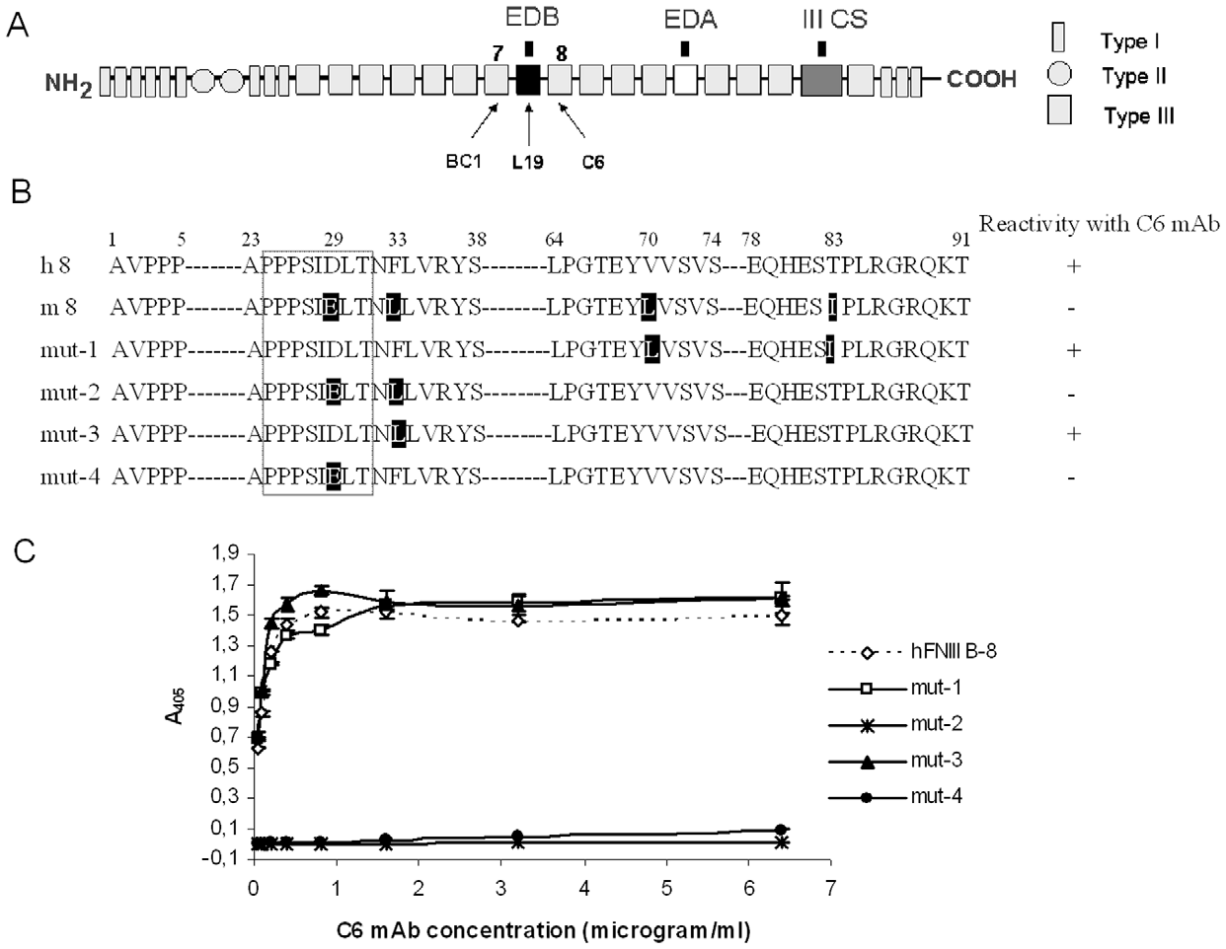
Reactivity of the mAb C6 with different FN fragments. ***A.*** Model of the domain structure of human FN subunit; the three different types of repeats and the specificity of the mAb C6 for the type III fibronectin repeat 8 are shown; ***B.*** Comparison of the amino acid sequence of human (h8) and mouse (m8) FNIII 8 domains; the differences in mouse FNIII 8 compared to human FNIII 8 are written in white letters on a black background. We generated four chimerical mutants of the human recombinant fragment FNIII B-8 (mut-1, mut-2, mut-3 and mut-4), substituting various amino acids of the human FNIII 8 with those of the mouse FNIII 8 sequence. The mouse residues introduced in the human sequence are in white letters on a black background. The loop B-C is enclosed in a box. The reactivity of each recombinant fragment with the mAb C6 is shown on the right. ***C.*** Reactivity in ELISA of various concentrations of the mAb C6 with human and chimeric FNIII B-8 recombinant fragments: C6 showed reactivity with human FNIII B-8, mut-1 and mut-3, but not with mut-2 and mut-4.

Fibronectins (FNs) are high-molecular-mass adhesive glycoproteins (for reviews see [1]–[3]) present in the extracellular matrix (ECM) and body fluids, constituted by a dimer composed of two subunits of about 250 kDa linked at the C-termini by two disulfide bonds. Each monomer consists of three types of repeating units: 12 FN repeats of type I (about 40 amino acids each), two type II (about 60 amino acids) and 15–17 type III (about 90 amino acids) (see Fig. 1A). Fibronectin mediates a wide variety of cellular interactions and is involved in a number of processes, such as cell adhesion, the establishment and maintenance of normal cell morphology, cell migration, growth and differentiation. It interacts with several other ECM and cell surface proteins, including collagen, heparin, fibrin, and cell membrane receptors. Finally, FN can be a ligand for numerous integrins, including the classic FN receptor alpha5-beta1 on the RGD sequence of repeat III-10 [4]. Fibronectins are the product of a single gene localized on chromosome 2 [5], but different isoforms arise from the alternative splicing of the pre-mRNA, a process that for some ECM proteins is modulated by cytokines and extracellular/intracellular pH [6], [7] in three sites: the type III connecting sequence (IIICS), a complete type III repeat, extra domain A (ED-A), and a complete type III repeat, extra domain B (ED-B) (Fig. 1A).

The last of these is a complete type III homology repeat of 91 amino acids, in which exon usage or skipping leads to inclusion or exclusion of these type III repeats. Our laboratory originally discovered the ED-B through comparative amino acid sequence analysis of proteolytic fragments of FNs from normal and transformed human cells, detecting an extra sequence that was preferentially included in FN from cultured transformed cells [8]. Independently and simultaneously, the ED-B was demonstrated by ribonuclease protection experiments on mRNA in rat and confirmed in human and was named EIIIB and EDII, respectively [9], [10]. The sequence of the ED-B is highly conserved in different species, having 100% homology in all mammalians thus far studied and 96% homology with a similar domain in chicken.

The generation of a murine monoclonal antibody (mAb), BC1 [11], selectively specific for FN containging the ED-B (B-FN), allowed the demonstration by immunohistochemistry that, while B-FN is undetectable in the tissues of healthy adults (with very rare exceptions, such as the recurrent physiological processes of tissue remodeling and angiogenesis occurring in the female reproductive system), it is, in contrast, abundant in fetal and neoplastic tissues. This antibody was widely used thereafter by different groups, and has allowed all of the early extensive immunohistochemical studies that have clearly established the outstanding presence of B-FN in different kinds of pathologies, including cancer and all angiogenesis-associated pathologies [12], [13], that B-FN is an excellent marker of angiogenesis [13], [14] and that the mAb BC1 could be used to selectively target cancer *in vivo* [15]. These results prompted the generation of high-affinity human recombinant antibodies specific for the ED-B for diagnostic and therapeutic purposes [16]–[22].

It has been previously reported that the insertion of the ED-B between the FN type III repeats 7 and 8 induces a conformational modification that unmasks a cryptic sequence and hinders others [23]. In fact, the B-FN specific mAb BC1 reacts with an epitope localized on FNIII 7 that is accessible only when the ED-B is included in the FN molecule; on the contrary, another monoclonal antibody, IST-6, reacts only with FN molecules lacking the ED-B. We recently described a new high affinity mAb, C6, which reacts with an epitope localized on FNIII 8 that is hindered when the ED-B is omitted but unmasked when the ED-B is present within the FN molecule [24]. We now report on the localization of this epitope on the B-C loop of FNIII 8.

## Results and Discussion

We previously reported on a mAb, C6, which reacts with an epitope within the FNIII 8 that is hindered when the ED-B is omitted but available when the ED-B is inserted within the molecule. However, C6 reacts only with human FN and not with mouse FN, thus suggesting that the epitope must be present in the sequences containing differences between human and mouse repeat FNIII 8. We compared the sequences of human and mouse FNIII 8 and, as is shown in Fig. 1B, they differ in only four amino acids, specifically at positions 29, 33, 70 and 83.

We generated chimerical mutants of human FNIII B-8 fragment substituting the human amino acids with the mouse amino acids and then tested the reactivity with the human specific antibody C6 by ELISA and immunoblot. ED-B is identical in mouse and human so the mutations were introduced only in the FNIII 8.

**Figure 2:**
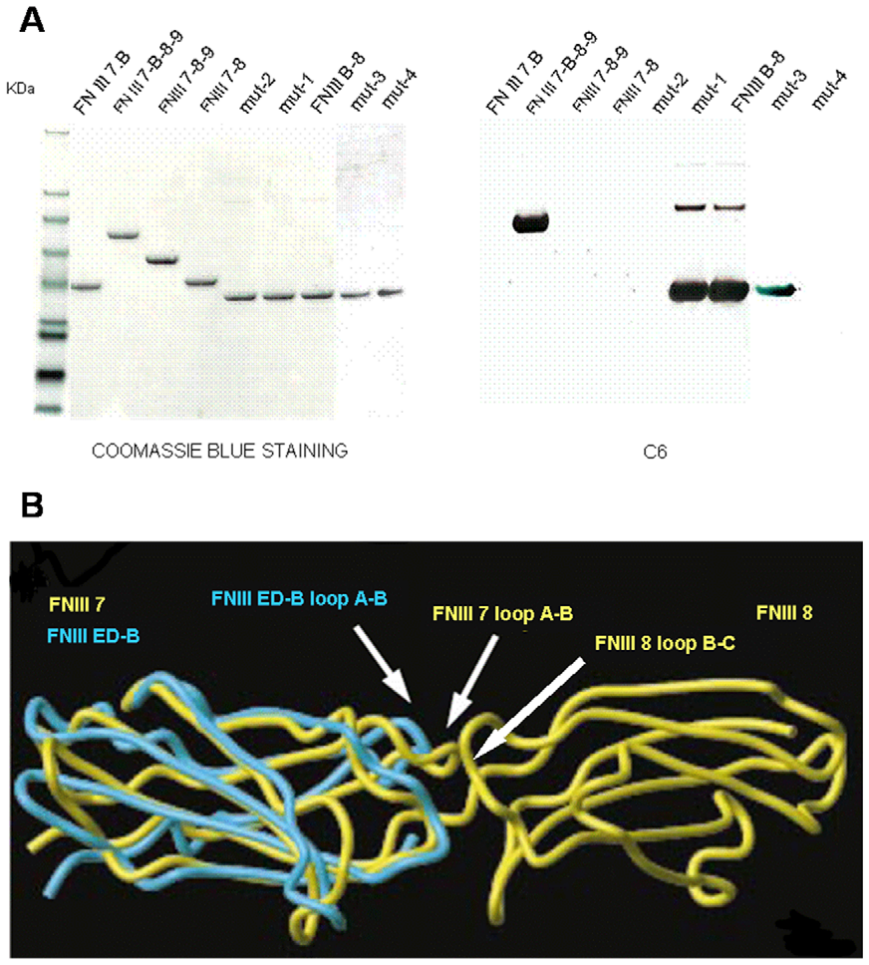
Westernblot of FN fragments using mAb C6 and structural comparison of interfaces FNIII 7/FNIII 8 and ED-B/FNIII 8. ***A.*** Coomassie blue staining of the human FN wild-type and mutated recombinant fragments FNIII 7-B, FNIII 7-B-8-9, FNIII 7-8-9, FNIII 7-8, mut-2, mut-1, FNIII B-8, mut-3 and mut-4 (left panel); reactivity in immunoblotting of the mAb C6 with the generated FN recombinant fragments: C6 reacted with FNIII 7-B-8-9, FNIII B-8, mut-1 and mut-3, but was negative with FNIII 7-B, FNIII 7-8-9, FNIII 7-8, mut-2 and mut-4 (right panel). ***B.*** Structural comparison of the interface between the ED-B/FNIII 8 and between the FNIII 7/FNIII 8 domains by superimposition of structures of the ED-B with FNIII 7. The FNIII 7 and ED-B loops A-B and the FNIII 8 loop B-C are indicated (adapted with permission from Fattorusso, R., et al., license number 2247580530397, dated Aug 14, 2009).

We first generated two mutants by substituting Val70→Leu and Thr83→Ile for mut-1 and Asp29→Glu and Phe33→Leu for mut-2. The recombinant proteins obtained from these two mutated cDNA showed that, in ELISA as well in immunoblot experiments, while mut-2 did not react with C6, mut-1 did not lose any immunoreactivity, thereby indicating that the epitope was localized among residues 29–33 of FNIII 8. Thus, we produced two new mutants, mut-3 and mut-4 with only one substitution each, Phe33→Leu and Asp29→Glu, respectively. The purified recombinant proteins obtained from these mutated cDNAs showed that mut-3 reacted, in ELISA experiments, with the C6 as well as the wild-type, while mut-4 completely lost its immunoreactivity (Fig. 1C). In immunoblot expriments, howewer, the reaction with C6 of mut-3 was weaker with respect to the completely human fragment. These results demonstrate that the epitope recognized by the monoclonal antibody C6 encompasses the Asp29 residue and the Phe33 may play a role in stabilizing it. This demonstrates that the epitope recognized by C6 is localized within the FNIII 8 loop B-C of which the Asp29 is definitively a part, and that this loop is hindered at the interface FNIII 7–8 but exposed at the interface FNIII B-8 (Fig. 2B).

Our conclusions concur with previously reported structural studies. The crystal structure of a human FN fragment of four type III repeats has shown a rod-like molecule with a highly variable relationship between the pairs of adjacent domains (type III repeats) [25]. The relative orientation of adjacent pairs of domains is regulated by interaction at the interdomain interfaces, which are largely composed of the loops located near the N and C termini of each domain [25], [26]. The type III homology repeat domains, independently of the degree of sequence homology, have highly conserved global folds and differ only in the loop regions, loops that are responsible for the biological activity of FN [25]. With regard to the FNIII 7–8 interface, reports have described that the inter-domain relationship is rigid and that the B-C loop of FNIII 8 is buried [25], [27], [28]. In fact, the interface FNIII 7–8 possesses a unique feature, namely an insertion of two amino acids in the A-B loop of FNIII 7 that influences the orientation of FNIII 7 and 8 by increasing the buried interface area [25] and that very likely accounts for the masking of the B-C loop of FNIII 8 (Fig. 2B). The insertion of the ED-B between FNIII 7 and FNIII 8 dramatically modified the inter-domain interfaces and interactions, with consequent conformational modifications including different domain orientation, the generation of new potential functional surfaces and the exposure of otherwise cryptic sequences [25]. In fact, it has been reported on differences in the location and conformation of the A-B, C-C′ and E-F loops in the ED-B compared to those of FNIII 7 [27]. These differences lead to the distinct buried inter-domain surface in FNIII 7–8 with respect to that of FNII B-8: the same authors show that the inter-domain linker between ED-B and FN8 buries a 416 Å^2^ surface, while the buried surface between FN7 and FN8 is 587 Å^2^, with an opening up of the surface area that exceeds 170 Å^2^ as a consequence of the inclusion of the ED-B [25], [27]. Our results are in line with these observations and experimentally demonstrate that the B-C loop of FN 8 is buried at the interface FNIII 7–8, while it is unmasked at the B-8 interface. Thus, the mAb C6, in addition to providing a new reagent for angiogenesis targeting, represents a new tool for the study of the biological activities of FN that are modified by the insertion of the angiogenesis-associated ED-B and, in particular, the potential biological functions of the B-C loop of the repeat FNIII 8.

## Materials and Methods

### Preparation of Wild-Type and Mutated Human FN Recombinant Fragments Containing the Type III Repeats ED-B (B) and 8, ELISA and Immunoblotting Assays

The cDNA encoding for human FNIII B-8 recombinant fragment, cloned into the bacterial expression vector pProEX-1 (Life Technologies, Gaithersburg, Maryland), was previously described [16]. The cDNA sequence of mut-1 was obtained by PCR from the cDNA of human FNIII B-8, using the primer forward TI-147 (5′-ctcgaattcaagaggtgccccaactcact-3′) and the primer reverse TI-148 (5′-ctcggatcctgttttctgtcttcctctaagaggtatgctctcatgttgttcgtagacactggagacactcactagatattc-3′), including the EcoRI and BamHI resctriction sites, respectively. The obtained sequence was EcoRI/BamHI digested and inserted into EcoRI/BamHI digested pProEX-1. The cDNAs of mut-2, mut-3 and mut-4, cloned into pProEX-1, were generated by site-directed mutagenesis from human FNIII B-8 cDNA and provided by Genscript Corporation (Piscataway, NJ, USA).

All PCR reactions were performed with high fidelity PWO DNA Polymerase (Roche Diagnostic, Basel, Switzerland) following the manufacturer's instructions. The restriction enzymes were from Roche Diagnostics.

The DNA constructs were used to transform DH5α competent bacteria cells and the recombinant wild-type and mutated FNIII B-8 fragments were purified from the bacterial lysate on Ni-NTA columns (Quiagen, Hilden, Germany) using the His6 tag appended at the N-terminus of the FN fragments, according to the manufacturer's instructions. The purified proteins were analyzed by SDS-PAGE under reducing conditions as previously described [21].

The recombinant fragments FNIII 7-B, FNIII 7-8-9, FNIII 7-B-8-9 and FNIII 7–8 were previously described [16].

The reactivity of the monoclonal antibody C6 [24] with recombinant wild-type and mutated FN fragments was assessed by ELISA and immunoblotting assays as previously described [24].
